# Virtual and In-Person Multiple Mini-interviews: A Comparison of Two Modalities in Regard to Bias

**DOI:** 10.1007/s40670-024-02142-5

**Published:** 2024-08-22

**Authors:** David D. Grier, Laurah Turner, Tracy J. Prichard, Andrea Oaks, David Nolan, Anisa S. Shomo, Dustin Dunlavy, Donald L. Batisky

**Affiliations:** 1 Department of Pediatrics, University of Cincinnati College of Medicine, Cincinnati, OH USA; 2 Division of Pathology, Cincinnati Children’s Hospital Medical Center, 3333 Burnet Avenue, MLC 1035, Cincinnati, OH 45229-3029 USA; 3 University of Cincinnati College of Medicine, Cincinnati, OH USA; 4 Department of Family and Community Medicine, University of Cincinnati, Cincinnati, OH USA; 5 Division of Nephrology, Cincinnati Children’s Hospital Medical Center, Cincinnati, OH USA

**Keywords:** Admissions, Virtual interview, Bias, Multiple mini interview

## Abstract

**Purpose:**

To examine the characteristics between virtual multiple mini-interview (vMMI) and in-person interviews (ipMMI) in regard to difference in performance between applicant-reported gender identity and racial groups.

**Methods:**

Retrospective multiple mini-interview (MMI) data from two vMMI interview cycles (2021 and 2022) consisting of 627 applicants and four ipMMI cycles (2017–2020) consisting of 2248 applicants. Comparisons were made between applicant subgroups including reported gender (male and female) and minority status (URiM and non-URiM). A three-way analysis of variance (ANOVA) was conducted to examine the effects of gender, URiM status, and interview modality (in-person vs. virtual) on MMI scores.

**Results:**

There were no overall significant differences between annual ipMMI and vMMI scores. A significant main effect of gender was observed, with females scoring higher than males overall. An interaction between gender and URiM status was also found. Although not statistically significant, when the MMI was virtual, URiM applicants on average scored higher than non-URiM applicants. In both the ipMMI and vMMI, URiM males tended to score lower than their non-URiM counterparts, though this difference was not statistically significant. URiM females tended to score higher than non-URiM females during the vMMI, and this difference was statistically significant.

**Conclusions:**

The switch to vMMI shows that there are no overall significant differences between the in-person and virtual formats; however, the finding that female URiM’s better performance in the virtual setting is novel. The cause of this finding is unknown but most likely reflects the complex interaction between race and gender. This insight requires future study and builds on the evidence that the MMI is an admissions tool to mitigate bias.

## Introduction

An interview is a required part of nearly all medical school admissions processes. With a few exceptions, prior to the COVID-19 pandemic, most interviews were conducted in person and consisted of either an unstructured one-on-one interview or a structured interview such as the multiple mini-interview (MMI). To protect both applicants and medical school staff during the COVID-19 pandemic, all medical school interviews went to a virtual format, starting either mid-cycle in 2019–2020 or at the beginning of the 2020–2021 admissions cycle[1, 2]. The transition required either the use of existing video conferencing technologies (e.g., Zoom, Microsoft Teams), the creation of an in-house system, or the purchase of a virtual interview system from a third-party vendor. Virtual interviews for medical school admissions were not unknown prior to the COVID-19 pandemic. A study published in 2018 placed the number of virtual interviews at US allopathic medical schools at 10% [3]. Virtual interviews for graduate medical education (i.e., residency and fellowship) were already being used by many programs prior to the pandemic [4]. In May of 2022, the Association of American Medical Colleges (AAMC) recommended the continuation of virtual residency interviews [5]. Several studies performed by residency and fellowship programs reported satisfaction with the virtual interview format [6–8]; however, some surveys found that in-person fellowship interviews were preferred by a majority applicants since they thought they could better evaluate a program’s facilities and meet faculty [9, 10].

Despite the associated costs and extra time required to learn, create, and/or purchase a system, several advantages seemed to emerge, for both the applicant and the medical school. Personnel performing interviews enjoyed greater flexibility, and there were possible cost savings for the institution [11, 12]. For the applicant, costs associated with travel, lodging, and time off from work decreased significantly or disappeared entirely. Lowering the financial barriers is believed to provide a more equitable medical school admissions process and greater access to medical school programs, including those that may have been out of reach for some applicants [13]. Decreasing the environmental impact of travel have also been seen as a reason to conduct virtual interviews [14, 15]. For these reasons, the AAMC has recommended that medical schools use virtual interviews with an optional in-person visit after acceptance [16].

Despite the potential advantages for the applicant, there were concerns about virtual interviews. One of the most concerning was exacerbating existing implicit and explicit biases that disproportionately affect applicants have been unrepresented in medicine (URiM) [17–19]. There were also concerns for new sources of potential bias including decreased access to adequate computer resources and Internet access, the difficulty in some facial recognition software recognizing darker skin, and the potential negative effect on interviewers observing applicants’ personal home or work environments seen through the video feed [17, 20].

Few studies in the pre-COVID-19 era directly compared virtual and in-person medical school interviews, and only one used virtual MMI (vMMI) as the interview format [21]. The University of New Mexico School of Medicine described their experience using a video-based platform in the pre-COVID-19 era but used a semi-structured interview format [3]. Several medical schools, both in and outside the USA, have published their experience of switching from in-person MMI (ipMMI) to vMMI, and none of them commented on the differences between the URiM applicants and the rest of the interview pool [11, 22–27]. Cork looked at Zoom vMMI and ipMMI and found the delivery of the MMI had comparable scoring [24]. Hammond and colleagues compared a synchronous vMMI using Zoom and their pre-COVID ipMMI for pharmacy school admissions and found that the vMMI was a reliable alternative to the ipMMI [28]. These forementioned studies did not specifically investigate race or gender as potential differences in the vMMI and ipMMI performance. This lack of comparison between vMMI and ipMMI performance across demographic factors is surprising given well-established literature on interview and rater bias and several articles describing how bias may manifest differently in the virtual setting [17, 18, 29–31]. This study describes a completely automated, synchronous MMI system using the same rater user interface for both vMMI and ipMMI with special attention paid to differences between different populations of interviewees.

## Methods

**Fig. 1:**
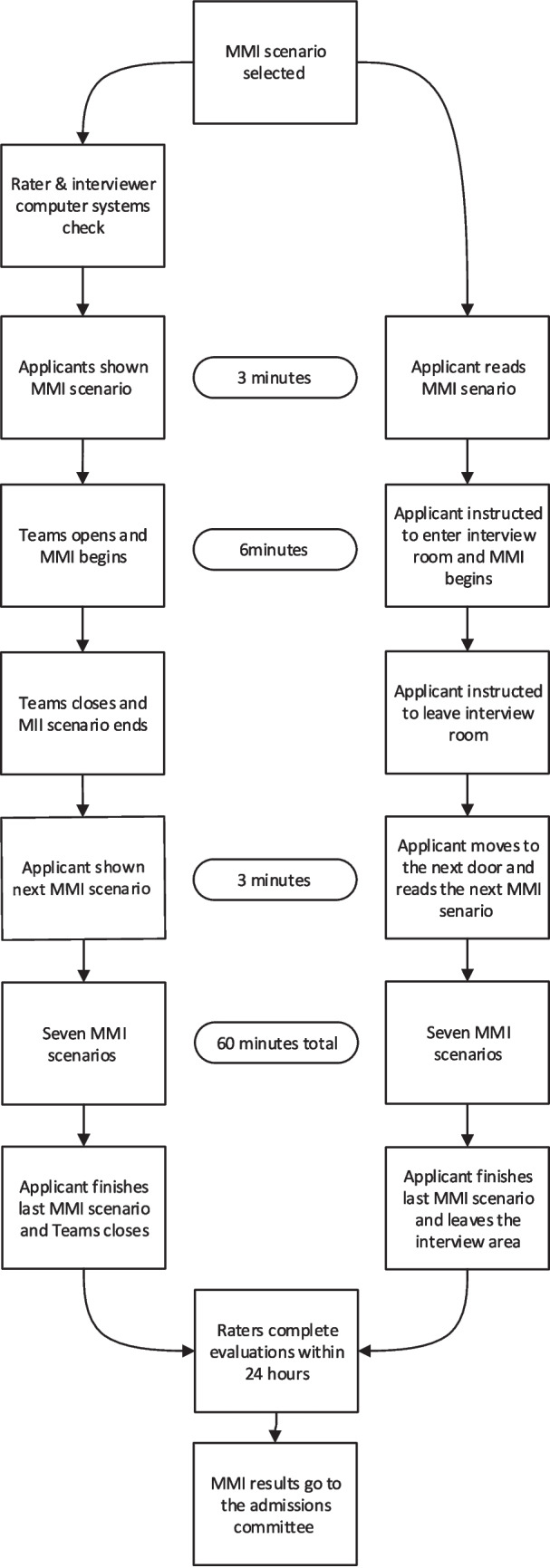
Comparison of in-person and virtual multiple mini-interview day sequence

The vMMI was developed using .NET (.NET Foundation, Richmond, WA) and integration with Microsoft Teams web version (Microsoft Corporation, Richmond, WA). An in-house developed student information system, MedOneStop, was used as an admissions interface for applicants, admissions staff, and raters. Both the vMMI and ipMMI had applicants rotate through eight stations, each with a different rater (interviewer) (Fig. 1). The same bank of MMI scenarios were used in both the ipMMI and vMMI. Scenarios that required interaction between two applicants were not used in the vMMI. Actors, either members of the community or medical students, were used in the ipMMI, but during the vMMI, the rater assumed the actor’s role. The raters were recruited primarily from the faculty, staff, and medical students at the University of Cincinnati College of Medicine (UCCOM) and Cincinnati Children’s Hospital Medical Center. First-time raters were required to attend a 1-hour online training session in addition to a general admission’s training session.

There were usually two vMMI sessions per interview day. To avoid common pitfalls such as blocked pop-ups, camera, and microphone issues, applicants and raters were required to complete a computer and video systems check within 24 hours of a vMMI. Raters were instructed to enter “Rater” as their name for anonymity. A photo of the student was provided on-screen for applicant reference and identity verification. The raters were blinded to the applicant’s American Medical College Application Service (AMCAS) and UCCOM secondary applications.

Each group (raters and applicants) met in separate initial Teams meetings with the admissions staff prior to the start of the vMMI to verify attendance and system operation. After the initial meeting and the official time began, applicants were shown their first station scenario for a set period (3 min). At this point, the applicant’s and rater’s browser would automatically open a Teams window for the interview to begin (6 min). At the end of the interview, the windows closed automatically, and the applicant was shown their next scenario (3 min). During this 3-min interval, the raters had the option to evaluate and assign a score to the applicant. After this period, the next station interview automatically began. The cycle continued until all applicants had rotated through eight stations. The process was automatic and could not be stopped for any one applicant. If at any point during a virtual interview an applicant or a rater experienced technical issues, they could click a button to message for help from IT support.

The ipMMI took place in the UCCOM’s Simulation Center, and typically two MMI sessions were done per interview day. After meeting with the admissions staff, applicants waited outside an individual simulation room door. They had 3 min to review the MMI scenario that was attached to the door. Guided by prompts from an overhead intercom, the applicant entered the room, and the interview began (6 min). At the end of 6 min, the applicant was prompted to leave the room and move to the next door where they had 3 min to read the next MMI scenario. The process was automatic and could not be stopped for any one applicant.

The same MedOneStop rater interface was used in the vMMI and the ipMMI. Both MMI modalities were cored using a Likert scale with 5 being the highest score and 1 being the lowest. The raters were trained to score towards the mean. The raters had the option to evaluate and score the applicant during the three-minute interval or within 24 hours from the start of the interview. The rater’s scores were adjusted based on their overall scoring over the course of the interview cycle.

Virtual adjusted annual MMI scores from medical school applicants in the 2021 and 2022 admissions cycles (*N* = 627) were compared with ipMMI scores from 2017 to 2020 (*N* = 2248) (Table 1). Additional comparisons were made between applicant subgroups including reported gender applicant-reported gender identity (male and female) and minority status (URiM and non-URiM). We used the AAMC definition of URiM as “any U.S. citizen or permanent resident who self-identified as one or more of the following race/ethnicity categories (alone or in combination with any other race/ethnicity category): American Indian or Alaska Native; Black or African American; Hispanic, Latino, or of Spanish Origin; or Native Hawaiian or Other Pacific Islander” [32]. The applicants select their race/ethnicity when completing the American Medical College Application Service® (AMCAS®) application.

**Table 1:** Number of interviewed applicants by MMI method, gender, and URiM status

Year	MMI Method	Female	Male	Total	URiM	URiM Female	URiM Male
2017	ipMMI	271	296	567	94	46	48
2018	ipMMI	301	325	626	123	52	71
2019	ipMMI	303	329	632	163	74	89
2020	ipMMI	322	296	618	153	86	67
Total ipMMI		1197	1246	2443	533	258	275
2021	vMMI	275	275	550	165	92	73
2022	vMMI	352	316	668	170	89	81
Total vMMI		627	591	1218	335	181	154
**Total**		**1824**	**1837**	**3661**	**868**	**439**	**429**

*MMI* multiple mini-interview, *ipMMI*, in-person MMI, *vMMI* virtual MMI, *URiM* underrepresented in medicine

Comparisons were made between applicant subgroups including reported gender (male and female) and minority status (URiM and non-URiM). To analyze the data, we conducted a three-way analysis of variance (ANOVA) with gender, URiM status, and application year group (in-person vs. virtual) as independent variables and the adjusted MMI score as the dependent variable. This approach allowed us to examine main effects and interactions between these factors. While Likert scale data are ordinal, the large sample size and the tendency for scores to be distributed around the mean justify the use of parametric statistics in this case. Levene’s test was used to assess the homogeneity of variances across groups. The UCCOM institution review board approved this research (2021–1032), including waiver of consent and the minimal risk to participants.

## Results

The three-way ANOVA revealed several significant findings regarding the effects of gender, URiM status, and application year group on MMI scores (Tables 2 and 3). Examining main effects, we found a significant influence of gender (*F*(3, 3663) = 25.707, *p* < 0.001), with females (*M* = 0.066, SD = 0.417) scoring higher on average than males (*M* =  − 0.066, SD = 0.428). However, neither URiM status (*F*(1, 3663) = 0.063, *p* = 0.802) nor application year group (*F*(1, 3663) = 0.139, *p* = 0.710) showed significant main effects, indicating no overall differences between URiM and non-URiM applicants or between in-person and virtual MMI scores.

**Table 2:** Descriptive statistics for adjusted MMI scores by gender, URiM status, and application year group

Gender	URiM	App Yr (group)	Mean	Std. seviation	*N*
F	N	In-person	0.065	0.419	939
F	N	Virtual	0.048	0.393	446
F	Y	In-person	0.078	0.424	258
F	Y	Virtual	0.100	0.448	181
M	N	In-person	− 0.057	0.421	971
M	N	Virtual	− 0.055	0.419	437
M	Y	In-person	− 0.092	0.444	275
M	Y	Virtual	− 0.102	0.467	154
Overall			− 0.001	0.428	3675
*F* (Overall)			0.066	0.417	1824
M (Overall)			− 0.066	0.428	1837
N (Overall)			0.001	0.419	2803
Y (Overall)			− 0.003	0.452	868
In-person (Overall)			0.000	0.428	2450
Virtual (Overall)			− 0.002	0.428	1225

**Table 3:** Three-way ANOVA results for adjusted MMI scores

Source	Type III sum of squares	df	Mean square	*F*	Sig
Corrected model	17.905	11	1.628	9.111	.000
Intercept	0.787	1	0.787	4.406	.036
Gender	13.778	3	4.593	25.707	.000
URiM	0.011	1	0.011	0.063	.802
App Year group	0.025	1	0.025	0.139	.710
Gender * URiM	0.834	1	0.834	4.667	.031
Gender * App year group	0.031	2	0.016	0.087	.917
URiM * App year group	0.028	1	0.028	0.155	.694
Gender * URiM * App year group	0.102	1	0.102	0.570	.450
Error	654.421	3663	0.179		
Total	672.327	3675			
Corrected total	672.326	3674			

*R*-squared = .027 (adjusted *R*-squared = .024)

We observed a significant interaction between gender and URiM status (*F*(1, 3663) = 4.667, *p* = 0.031). URiM females tended to score higher (*M* = 0.087, SD = 0.434) compared with non-URiM females (*M* = 0.060, SD = 0.411), while URiM males tended to score lower (*M* =  − 0.096, SD = 0.452) compared with non-URiM males (*M* =  − 0.056, SD = 0.420).

We found no significant interactions between gender and application year group (*F*(2, 3663) = 0.087, *p* = 0.917) or between URiM status and application year group (*F*(1, 3663) = 0.155,* p* = 0.694). Additionally, the three-way interaction between gender, URiM status, and application year group was not significant (*F*(1, 3663) = 0.570, *p* = 0.450).

It’s worth noting that Levene’s test indicated unequal variances across groups (*F*(11, 3663) = 1.990, *p* = 0.026). While this suggests some caution in interpreting the results, the large sample size and the robustness of ANOVA to moderate violations of homogeneity of variance lend credibility to our findings.

## Discussion

Virtual interviews were being used extensively in job recruitment before the COVID-19 pandemic. A 2015 survey done by FutureStep, a recruitment process outsourcing and professional search company, found that 75% of companies used real time (synchronous) video interviews[33]. Despite the ubiquitous utilization of virtual interviews since the start of the COVID-19 pandemic, there is very little literature examining the role of bias specifically in virtual interviews. The AAMC introduced the Standardized Video Interview (SVI) as an online, asynchronous video interview designed to holistically assess applicants’ proficiency in two of the Accreditation Council for Graduate Medical Education (ACGME) competencies of interpersonal/communication skills and professionalism in the emergency medicine field [34]. After 4 years, the AAMC discontinued the SVI due to the lack of interest and the challenge of scaling the SVI to the multiple residencies [35]. A review of the data showed that SVI scores slightly favored Black applicants compared with white applicants and found no difference between Latino and Asian applicants compared to white applicants [36]. While female applicants did better than male applicants, there was no mention about how Black female applicants did as a separate category.

Our analysis revealed a significant main effect of gender on MMI performance, with females consistently outperforming males across both in-person and virtual formats. This finding aligns with previous research [17, 19, 37] and warrants further investigation into the underlying factors contributing to this gender difference in MMI performance.

The significant interaction between gender and URiM status provides new insights into the complexities of demographic factors in MMI performance. Notably, URiM females performed particularly well in the virtual MMI format, outscoring their non-URiM counterparts. This finding challenges concerns that virtual interviews might disadvantage URiM applicants and suggests that the virtual format may actually benefit certain demographic groups.

Contrary to some concerns raised in the literature, we found no significant main effect or interactions involving the application year group (in-person vs. virtual). This suggests that the transition to virtual MMIs did not systematically advantage or disadvantage any particular group, supporting the validity of virtual MMIs as an alternative to in-person interviews.

To our knowledge, this is the first study looking at within group and between group differences of a synchronous, virtual, and in-person MMI and how the two modalities affect applicant scoring between demographic groups such as race and gender. Female applicants performing better than their male counterparts in both MMI and non-MMI in-person interviews is well documented in the literature [38–40]. Other studies have reported that URiM status did not affect MMI performance [38, 41].

The results of the SVI and this study both showed that Black applicants did better than their white counterparts in these video interviews, although the SVI was asynchronous and our vMMI is synchronous. The underlying reason for this finding is uncertain. There is no literature delving into the reasons why Black females would have better performance in a virtual interview. It should be noted that in a large multiple national study of in-person MMI and traditional interviews, 4993 interviewees who underwent 7516 interviews, there was no association with URiM status and interview performance; however, these interviews were conducted in-person [38].

Several limitations should be considered when interpreting these results. First, while we used parametric statistics (ANOVA) with Likert scale data, this approach is justified by our large sample size and the tendency for scores to distribute around the mean. However, future studies might consider alternative statistical approaches specifically designed for ordinal data.

Second, Levene’s test indicated unequal variances across groups. While ANOVA is generally robust to moderate violations of homogeneity of variance, especially with large and balanced samples, this should be considered when interpreting the results.

Finally, this study was conducted at a single institution, which may limit the generalizability of our findings. Multi-institutional studies would be valuable to confirm these results across different settings and populations.

## Conclusions

The transformation from in-person multiple mini-interviews (ipMMI) to virtual multiple mini-interviews (vMMI) in medical school admissions amid the COVID-19 pandemic offered an unanticipated opportunity to examine biases and differences within a complex evaluation framework. Our study, conducted at the University of Cincinnati College of Medicine, stands as the first to describe and compare both vMMI and ipMMI modalities, with an emphasis on their effect on different demographic groups. Our study demonstrates that the transition from in-person to virtual MMIs did not introduce significant biases or alter performance patterns across demographic groups. The consistent gender effect and the complex interaction between gender and URiM status highlight the need for continued research into factors influencing MMI performance. These findings support the use of virtual MMIs as a viable alternative to in-person interviews, potentially offering a more accessible and equitable admissions process. Future research should focus on understanding the underlying mechanisms of these demographic differences and exploring ways to ensure fair assessment across all applicant groups.

## Data Availability

The data that support the findings of this study are not openly available due to reasons of sensitivity and privacy of the interviewees and raters.
